# 619. Fungal Species Causing Eumycetoma and Chromoblastomycosis in a Commercial Laboratory, United States

**DOI:** 10.1093/ofid/ofad500.685

**Published:** 2023-11-27

**Authors:** Dallas J Smith, Jeremy A W Gold, Shawn R Lockhart, Kaitlin Benedict

**Affiliations:** Mycotic Diseases Branch, Centers for Disease Control and Prevention, Atlanta, Georgia; Centers for Disease Control and Prevention, Atlanta, Georgia; Centers for Disease Control and Prevention, Atlanta, Georgia; CDC, Atlanta, Georgia

## Abstract

**Background:**

The World Health Organization (WHO) designates the implantation mycoses eumycetoma and chromoblastomycosis as Neglected Tropical Diseases. These infections usually develop after traumatic skin inoculation and primarily occur in resource-limited countries in tropical or subtropical areas. They are difficult to diagnose, may require prolonged antifungal therapy and surgery if not diagnosed early, and cause substantial stigma and morbidity. Understanding the organisms causing these diseases and their epidemiology might help inform best clinical practices in the United States.

**Methods:**

We analyzed Labcorp (a major U.S. commercial laboratory) data sent to CDC’s National Syndromic Surveillance Program. We identified results from cultures ordered during 3/1/2019–3/1/2023 involving fungi known to cause eumycetoma and chromoblastomycosis. Nail and scalp specimens were excluded. We examined patient demographic characteristics, ordering clinical specialty, and species.

**Results:**

We identified 33 eumycetoma and 119 chromoblastomycosis results. For eumycetoma, most patients were aged ≥ 50 years (mean=56, interquartile range 42-69), male (64%), and diagnosed in the South census region (52%). Dermatology ordered 21%; family practitioners ordered 18%. *Scedosporium apiospermum* species complex was the most common species (94%). Another species identified was *Exophiala jeanselmei.* For chromoblastomycosis, 31% of results were from patients 45–64 years old; 29% were ≥ 65. Most were male (53%), and results were evenly distributed among the Northeast (33%), South (34%), and West (31%) census regions. Most cultures were ordered by dermatologists (70%). Fifty-two percent of results were *Aureobasidium pullulans*, 24% were *Exophiala* (undetermined species), and 12% were *Exophiala dermatitidis*. *Phialophora verrucosa, Fonsecaea pedrosoi*, *Exophiala jeanselmei,* and *Exophiala xenobiotica* accounted for the other 13%.

**Conclusion:**

Although rare, clinicians should be aware that eumycetoma and chromoblastomycosis can occur in U.S. patients seeking treatment for subcutaneous skin infections. Identifying the epidemiology of causative organisms can inform evidence-based practices for diagnosis and treatment.

**Disclosures:**

**All Authors**: No reported disclosures

